# Supplementary material for: Medical Misinformation in AI-Assisted Self-Diagnosis: Development of a Method (EvalPrompt) for Analyzing Large Language Models
Source: JMIR Form Res. 2025 Mar 10;9:e66207. doi: 10.2196/66207 (PMC11913316; doi:10.2196/66207)
Supplement: Multimedia Appendix 1 [file formative-v9-e66207-s001.docx]

## ChatGPT-4.0 Model Selection and Parameters

In this study, the ChatGPT-4.0 responses were obtained between March and April 2024 using the *gpt-4* model. The parameters used for the model are summarized in Table S1.

**Table S1.** Model parameters used to produce the ChatGPT-4.0 responses. In particular, the *gpt-4* model was used to aggregate the initial responses and the responses from the ablation study.

| *Parameter Name* | *Parameter Value* |
| --- | --- |
| model | gpt-4 |
| max_tokens | 2048 |
| n | 1 |
| stop | None |
| temperature | 0 |
| top_p | 1 |
| frequency_penalty | 0 |
| presence_penalty | 0 |
