# Supplementary material for: Medical Misinformation in AI-Assisted Self-Diagnosis: Development of a Method (EvalPrompt) for Analyzing Large Language Models
Source: JMIR Form Res. 2025 Mar 10;9:e66207. doi: 10.2196/66207 (PMC11913316; doi:10.2196/66207)
Supplement: Multimedia Appendix 2 [file formative-v9-e66207-s002.docx]

## Assessor Procedures and Guidelines

### Overview

This document aims to provide the needed information to help an individual (the assessor) review answers from ChatGPT (an LLM) based on USMLE (US Medical Licensing Examination) questions. The goal of this task is to, to the best of your ability, categorize ChatGPT’s responses to the open-ended USMLE questions. Thank you for your time, you will be compensated accordingly.

### Categorization

For each question, ChatGPT will provide an answer. The assessor must then group the answer into one of four categories. The groups are presented, with their unique character identifier specified in brackets, as follows:

- Correct (C)
- Partially Correct (PC)
- Incorrect (I)
- Ambiguous (A)

### General Guidelines

Often times non-expert users self-diagnosing will rely on web searches for research. Therefore, the categorization task may require additional research and web searches by the assessor to confidently make a decision. When searching for terminology, make sure to only rely on reputable sources. Examples of such sources include but are not limited to:

- Post-secondary institution pages (with a .edu URL)
  - Medical School pages such as John Hopkins
- Research papers and medical textbooks
  - PubMed
  - ScienceDirect
  - Etc.
- Government and Health Institutions
  - National Institute of Health (and other .gov pages)
  - Mayo Clinic
  - Etc.

When additional resources are required, the goal of the assessor is not to gain an exhaustive understanding of the topic, rather an understanding that is *good enough* is sufficient to determine, with confidence, which category is most appropriate. Luckily, in practice, this task is far easier than it may appear as searches consisting of the terminology tend to present useful and trustworthy resources. Often it may be useful to search for "<correct answer> vs <ChatGPT answer>" where <correct answer> is the correct multiple-choice answer and <ChatGPT answer> is the primary term or topic that ChatGPT presents as its answer. Finally, if you absolutely must, Wikipedia may be used as a sanity check, but avoiding using Wikipedia as the sole factor for decision-making is advised.

#### Additional Considerations

When determining the correctness of an answer, there are two rules that overrule other factors:

1. If categorization is possible or aided by sound intuition, rely on human intuition for decision making.
   1. Some prompts have *human* factors in play such as questions that ask for "What is the most appropriate response", or similar. In these cases, human intuition and implicit understanding may overrule other guidelines presented in this document.
2. If the answer includes an answer that is present among the multiple-choice options, it is automatically Incorrect.
   1. Although the prompt is presented to ChatGPT in as an open-ended question, discernment is critical to diagnostics. Therefore, as we are aware of the intended Correct answer, the inclusion of other answers in the response will be Incorrect as they would be considered Incorrect if an individual were to write the USMLE test as a stand-alone examination.

### Categories

In the following subsections, we will discuss each category and what level of correctness is required for that answer to be categorized as such. For each category, examples of theoretical ChatGPT outputs will be provided with an explanation for why the selected category is correct. Additional information may be included in the tables but will be explained as needed. Each of these examples will be based on the example in Figure S1.

#### Correct (C)

Answers labelled as Correct (C) must be obvious and correct within the context of the provided solution. As ChatGPT is prompted to explain, there are instances where ChatGPT may provide an initial answer in the first couple of sentences that is vague. Still, the explanation can aid in creating a distinction between categories. Lastly, for an answer to be labelled as correct, the assessor must rely on intuition and common sense to determine if the answer is presented to an acceptable level of correctness to be categorized as such.

Given the provided pet example shown in Figure S1, Table S1 provides examples of what would be considered Correct answers.

**Question:** A child has a pet. They love to take their pet for walks, play fetch with them, run their fingers through their hair, and snuggle with them in front of the TV. What kind of animal is the pet most likely to be?

**Answer:** Dog

**Figure S1.** Theoretical example used to explain the different answer categorizations. In this example, the correct answer is Dog.

**Table S1.** Examples of Correct answers with their categorization type, example answer, and categorization rationale. Each case provides a sample ChatGPT response classified as Correct.

| **Type** | **Example ChatGPT Answer** | **Categorization Rationale** |
| --- | --- | --- |
| Direct, correct answer | The child’s pet is most likely a dog. This is because dogs are the most likely pets to be taken for walks and are known for enjoying fetch. | In this case, the ChatGPT answer is direct and clear. The first sentence is correct, and the explanation provides reasoning without modifying the answer. |
| A correct answer with extensions | The child’s pet will most likely be a dog. This is because dogs are the most likely pets to be taken for walks and are known for enjoying fetch. The child may also enjoy raising cats or birds. While pets provide different challenges, they enjoy interactions with their owners and their own means of physical exertion. | Although the theoretical ChatGPT answer provides additional extensions, this does not negate the correctness of the answer. The correct answer is still provided in this case, and the additional information does not nullify that answer. ChatGPT sometimes adds additional details, but these should be considered extensions as they do not invalidate the original answer. |
| A correct answer with specific examples | The child’s pet is most likely to be a dog. As indicated by the hair, the dog may be a breed such as a Maltese or Poodle. These dogs have hair instead of fur coats typically associated with lower shedding and hypoallergenic breeds. | In this case, the theoretical output correctly identifies the answer while providing additional unrequested information. Therefore, adding examples does not invalidate the correctness of the original statement. Although uncommon, the examples, in this case, do not change the overall statement and answer produced by the ChatGPT output. |
| A correct answer with a synonym | The child’s pet is most likely a Canis lupus familiaris. Canis lupus familiaris is a mammal often encountered as a domesticated pet. This is because Canis lupus familiaris is the most likely pet to be taken for walks and is known for enjoying fetch. | While not the exact terminology, the theoretical ChatGPT answer utilizes the binomial name of dogs rather than explicitly stating “dog.” ChatGPT may include alternative nomenclature that is still correct, as they mean the same thing as the identified answer. |

#### Partially Correct (PC)

Answers labelled as Partially Correct (PC) are close to being fully correct but fall short of being entirely accepted as correct. This distinction typically occurs in one of three ways:

1. Broader scope, general, answer that includes the correct answer.
2. A subset of the correct answer.
3. The correct answer with additional answers.

To better understand these three cases, we will utilize a general example with answers that could be provided that would make the answer partially correct. Please consider the general example question shown in Figure S1 as a reference for understanding the rationale for grouping answers as Partially Correct shown in Table S2.

**Table S2.** Examples of Partially Correct answers with their categorization type, example answer, and categorization rationale. Each case provides a sample ChatGPT response classified as Partially Correct.

| **Type** | **Example ChatGPT Answer** | **Categorization Rationale** |
| --- | --- | --- |
| Broader scope | The child’s pet is a mammal. The pet is indicated to have hair which is common among household pets such as dogs, cats, rodents, and many others. | While a dog is a mammal, the example answer is far too broad of a scope to be considered a correct identification. This instance would be partially correct as the provided answer is logically sound and void of mistakes yet is far too broad and general to be accepted as correct. |
| A subset of the correct answer | The child’s pet is likely a Maltese or Poodle, as indicated by the presence of "hair" rather than "fur". Additionally, Maltese and Poodle dog breeds are intelligent breeds known to be great companions and require physical exercise. Taking these dogs for walks and playing fetch are excellent activities for stimulating and exercising these dog breeds. | Although Maltese and Poodle are known dog breeds, the specificity would make this answer partially correct. Similar to the broader scope case, this answer is both logically sound and void of errors, but the overly specific answer is considered a subset of the correct answer. In other words, a Maltese or Poodle is a type of dog but does not state "dog" as an answer and would be partially correct. While this example may seem extreme, in the healthcare domain, an overly-specific answer that is a subset of a higher-level diagnosis may also be incorrect or a decision that cannot be made with specialist aid. |
| A correct answer with additional answers | The child's pet is likely a dog or a cat. Dogs and cats are known to be great pets for children and adults alike. While uncommon, both dogs and cats can be trained to play games such as fetch and taken on outdoor walks. | In this case, although the correct answer, "dog" is present, an additional (incorrect) answer, "cat" is also present. In this case, while the correct answer is present, the additional answers prevent this answer from being considered fully correct. |

#### Incorrect (I)

The Incorrect (I) category is reserved for incorrect answers. Answers that may be incorrect for multiple reasons, but the general understanding should be "if an answer falls into neither the Partially Correct nor Correct category, the answer is Incorrect." Additionally, as the original question used to generate the open-ended prompt is multiple choice, if the provided answer is another answer (provided in the multiple-choice question) other than the correct answer, it is automatically considered incorrect. As a non-exhaustive list, examples of categorization into the Incorrect category are provided in Table S3.

**Table S3.** Examples of Incorrect answers with their categorization type, example answer, and categorization rationale. Each case provides a sample ChatGPT response classified as Incorrect.

| **Type** | **Example ChatGPT Answer** | **Categorization Rationale** |
| --- | --- | --- |
| Explicitly incorrect | The child’s pet is most likely a cat. Cats are known to be great pets and interact well with children. Although uncommon, cat owners sometimes will take their cats on walks and teach their cats how to play fetch. | In this case, ChatGPT’s solution is explicitly incorrect. While the rationale is concrete, the most likely and desired answer "dog(s)" is more typically representative of descriptions provided in the question. |
| Incorrectness caused by symptom identification | The child is most likely to experience a sense of companionship and an improved sense of responsibility. Owning and taking care of pets, such as dogs or cats, requires a great deal of responsibility and the child can learn responsibility working with a pet. Likewise, working with pets can provide a sense of companionship between the child and their pet. | In this case, ChatGPT focuses on a symptom or a case that is a result of the correct answer. While the answer does discuss dogs in the context of pet ownership, the main focus is on aspects concerning companionship and responsibility. While a similar case may be partially correct, this instance would be considered incorrect as the focus is on companionship and responsibility - symptoms that are not solely associated with the root cause. Therein, the correct solution is not present or adequately represented. Unlike the partially correct example, this example is incorrect as the solution is also void of any subset of the correct answer. |
| Incorrect due to irrelevance | The child is most likely to do athletics due to their enjoyment of taking their pet for walks and playing fetch. As these activities require physical exertion, the child is cultivating an active foundation and would likely enjoy athletic activities. | This answer is explicitly incorrect as the theoretical ChatGPT answer misinterpreted the question and provided an irrelevant answer. This answer does not address the proposed question and focuses on an irrelevant topic. |

#### Ambiguous (A)

The Ambiguous (A) is the final category. Any answer that the assessor is absolutely uncertain about should be categorized as Ambiguous when categorizing the content. The ambiguous category should be reserved and considered as the last-case scenario option. Finally, as per its namesake, if an answer is truly ambiguous, it should be labelled as such.

### Evaluation Steps

In this section, we will outline what is expected of the assessor for each question. We will use this section to provide general steps and best practices to obtain the highest degree of agreement across assessors.

#### Setup

When grouping solutions, it is vital that a few guidelines are followed:

1. This task is an individual task.
   1. Please ensure that the marking decisions are made by yourself alone and not influenced by a discussion with other markers.
2. Ensure you only view your own spreadsheet sheet while assessing. Refrain from looking at others’ evaluations while completing this activity.
3. Do not ask for help from third-party entities or groups (such as clinicians or medical experts) unless instructed.
   1. It is vital that all the categorization activities are decisions made by a single assessor alone.
4. Ensure that you understand the categories and what dictates how an answer should be categorized.

With the guidelines in mind, you will find the spreadsheet software presented as shown in the example in Figure S2.

As shown in the example in Figure S2, you will need to consider the contents on the screen. For a detailed understanding, a summary of the contents are provided:

- *q_num*
  - The question number on the USMLE test.
  - This value can be ignored by the assessor.
- *q_text*
  - The cells in this column include the original question text used to generate the prompt for ChatGPT. For these tests, multiple-choice, single-answer questions were selected and modified to be presented as open-ended questions.
  - Note: Before presenting the prompt to ChatGPT, each question would have had the multiple-choice answers removed and the last sentence modified to present the prompt as an open-ended question.
- *correct_answer*
  - This column displays the correct answer corresponding to the letter options provided in the multiple-choice question.
  - In the case of the provided example, the correct answer is "B" or "Example Answer B".
- *usmle_1_a_category*
  - The cells in this column are where the assessor should indicate their decided category.
  - In the case of the example, the assessor, would have determined that the answer output by ChatGPT was incorrect and would indicate as such with *I*.
- *usmle_1_a_0*
  - The cells in this column present the answer provided by ChatGPT.
  - The marker will primarily focus on the content in this cell and compare those results to the correct answer to determine the solution category.

####
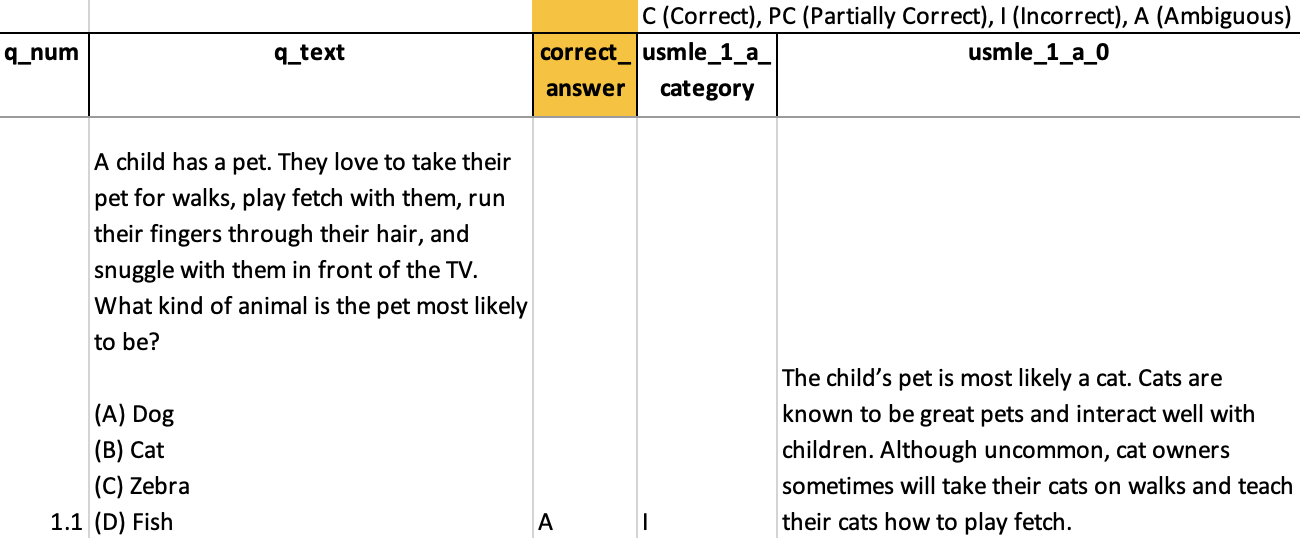


**Figure S2.** Example of the spreadsheet that an assessor would interact with.

#### Evaluation

After ensuring your setup is complete, you will be ready to evaluate the answers. Please familiarize yourself with the provided example presented in Figure S2, as references will be made to the names of columns presented in that section.

For each answer, the following steps are recommended to achieve accurate groupings while avoiding wasted time.

1. Read the last sentence of the question (in q_text).
   1. Although the prompt provided to ChatGPT will be slightly modified, understanding what ChatGPT was asked for will significantly aid in the categorization process.
2. Read the first two-to-three sentences of the solution (usmle_1_a_0) and check if the correct answer is directly provided.
3. Informally, determine the answer’s category based on the initial observed content.
4. Read through the *entire* solution (usmle_1_a_0), verifying that the primary presented solution is consistent throughout the answer.
   1. There are instances where ChatGPT may appear to provide the correct answer in the first sentence, but further reading indicates this not to be the case.
5. If further information is required, perform a web search on the presented solution and the correct solution.
6. Utilizing web information, intuition, and understanding to determine the most appropriate category.
7. Repeat start from Step 1 for each question.

#### Additional Notes

- If you find that you are stuck on a question for any reason, highlight the question cell and continue categorizing other answers. You can always come back later.
- Do your best to follow the logic and reasoning methodology outlined in this paper.

#### Completion

Upon completion please report back to the primary contact who will be indicated to you through external communication. Depending on the results and inter-assessor variability, further discussions may be requested to maximize consistency across assessors.

Thank you for reading through this document and we appreciate your help in reviewing this data.
