# Supplementary material for: Medical Misinformation in AI-Assisted Self-Diagnosis: Development of a Method (EvalPrompt) for Analyzing Large Language Models
Source: JMIR Form Res. 2025 Mar 10;9:e66207. doi: 10.2196/66207 (PMC11913316; doi:10.2196/66207)
Supplement: Multimedia Appendix 3 [file formative-v9-e66207-s003.docx]

## ChatGPT-3.5 Categorization Results

As the evaluation process was also conducted on ChatGPT-3.5 (GPT-3.5) using the parameters provided in Table S1, a comparative analysis between GPT-3.5 and GPT-4.0 could be completed proving that GPT-4.0 is a substantial improvement. Table S2 displays the aggregated categorization results for GPT-3.5 for both Experiment 1 and Experiment 2. Three assessors categorized the responses for the two experiments. Experiment 1 used the same initial 94-question dataset, while Experiment 2 used a 56-question dataset that spanned 9 unique questions. The individual categorizations for Experiment 1 and Experiment 2 are provided in Figure S1 and Figure S2, respectively. Most of the responses for Experiment 1 were categorized as Incorrect, while most of the Experiment 2 responses were categorized as Correct. This means that GPT-3.5 answers that were initially Correct, continue to be Correct even after sentences are removed.

**Table S1.** Model parameters used to produce the ChatGPT-3.5 responses. In particular, the *text-davinci-003* model was used to aggregate the initial responses and responses from the ablation study.

| *Parameter Name* | *Parameter Value* |
| --- | --- |
| model | text-davinci-003 |
| max_tokens | 2048 |
| n | 1 |
| stop | None |
| temperature | 0 |
| top_p | 1 |
| frequency_penalty | 0 |
| presence_penalty | 0 |

**Table S2.** Number of identical categorizations amongst all individuals evaluating GPT-3.5 for the two experiments. Experiment 1 had 94 questions in total, with 55 of the questions being categorized the same among the assessors. Similarly, Experiment 2 had 56 responses in total spanning 9 unique questions, with 48 of the questions being categorized the same among the assessors.

|  | *Experiment 1* | *Experiment 2* |
| --- | --- | --- |
|  | Non-expert | Non-expert |
| Correct | 9 | 36 |
| Partially Correct | 12 | 5 |
| Incorrect | 34 | 7 |
| Ambiguous | 0 | 0 |
| Overall Agreement | 55 | 48 |


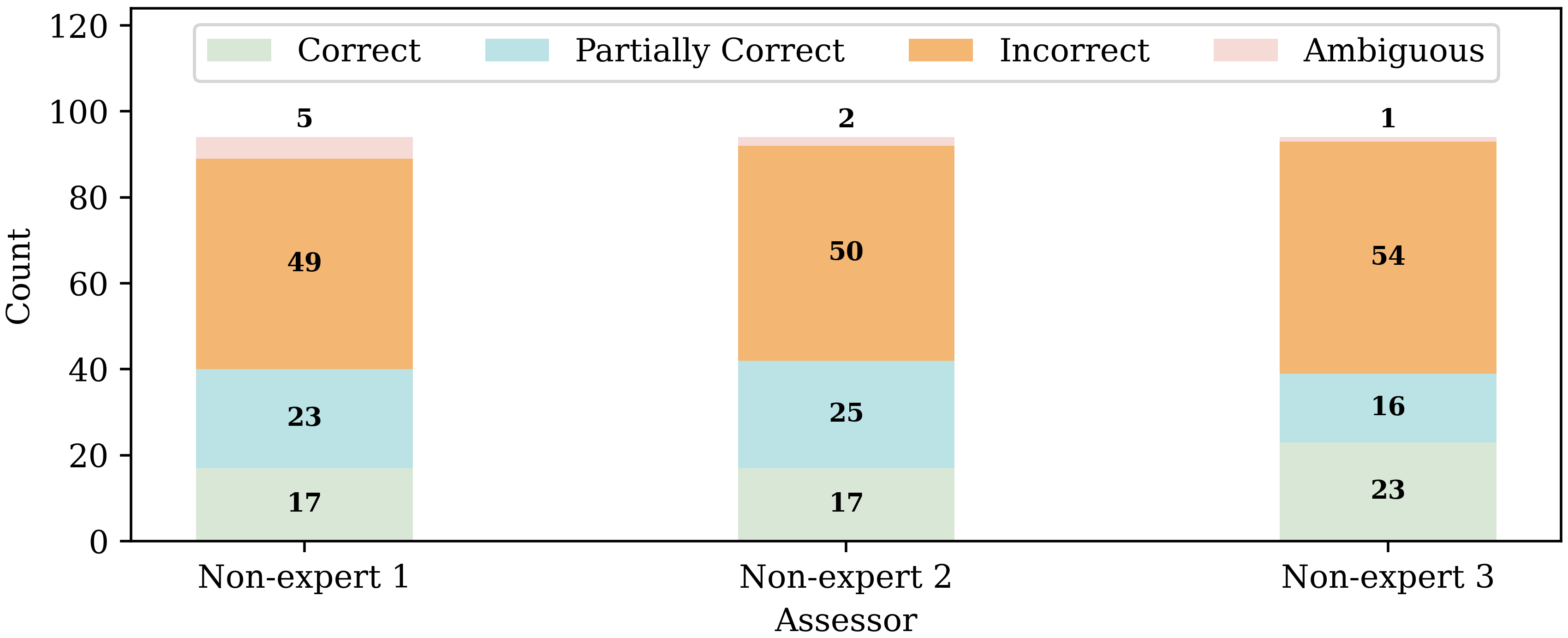


**Figure S1.** Experiment 1: Individual categorization for the non-expert assessors for GPT-3.5. The responses are mainly categorized as Incorrect.


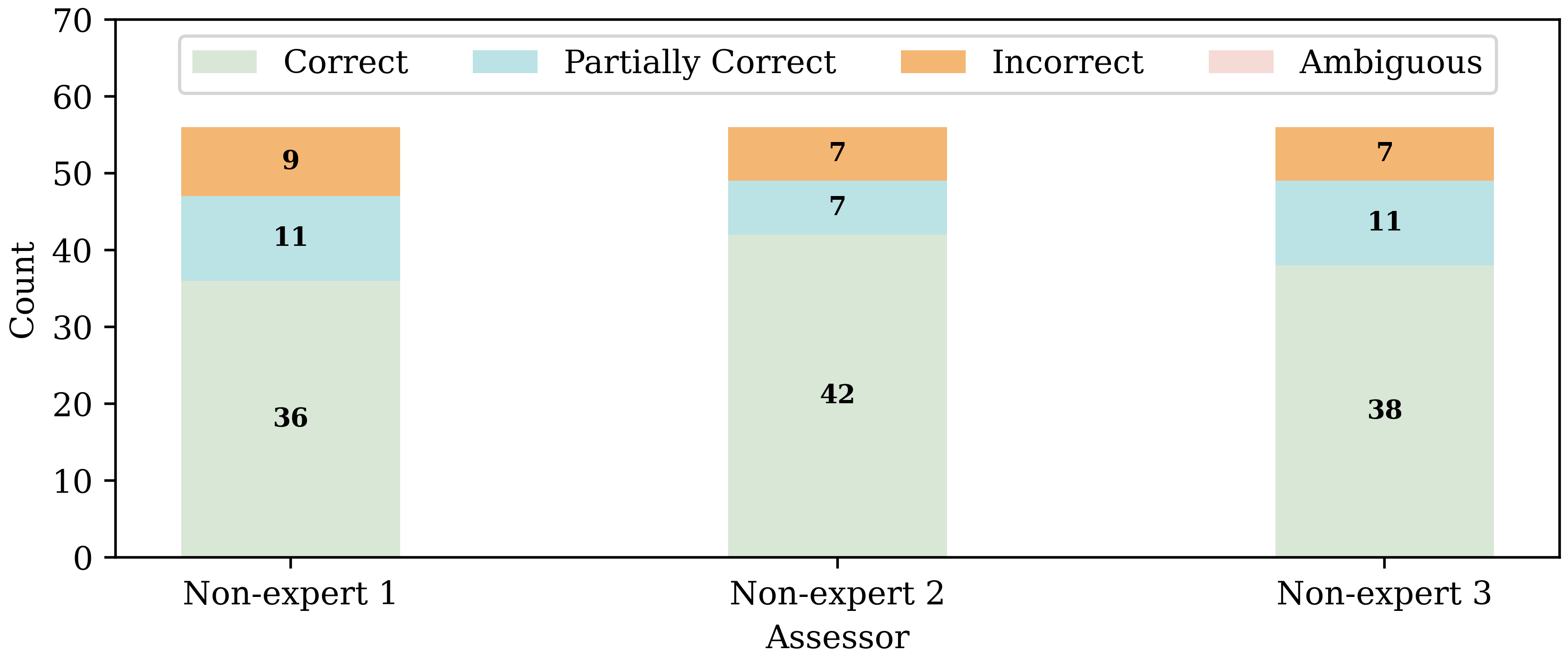


**Figure S2.** Experiment 2: Individual categorization for the non-expert assessors for GPT-3.5. Out of the 56 questions, the assessors on average categorized 39 of the questions as Correct.

Comparing the number of Correct responses for the GPT-3.5 non-experts to the GPT-4.0 non-experts, as provided in Table S3, nine of the responses were categorized as Correct for GPT-3.5, while 33 of the responses were categorized as Correct for GPT-4.0. Consequently, by only considering the Correct categorizations, GPT-4.0 is automatically a 266% improvement compared to GPT-3.5. Using the t-test statistic [40] to further evaluate the correctness of the two models, as defined in Equation 1, GPT-4.0 is an improvement compared to GPT-3.5. The test compares the mean of two groups to determine if there is a statistically significant difference between them.

$$t_{ij}^{z}=\frac{\bar{x}_{i}-\bar{x}_{j}}{\sqrt{\frac{s_{i}^{2}}{n_{i}}+\frac{s_{j}^{2}}{n_{j}}}}$$

At a significance level of $0.10$, $t=7.78$ with a corresponding $p=0.0015$. Therefore, since $p<0.10$, there is a statistically significant difference between the two models. In GPT-3.5, assessors noted brief yet repetitive responses suggesting incorrect comprehension and overconfidence when stating incorrect recommendations. This concerning tendency increases the risk of spreading medical misinformation and misdiagnoses. Comparably, GPT-4.0 has significantly improved in answering open-ended medical questions by providing detailed responses supported by scientific reasoning.

**Table S3.** Overall response categorization between GPT-4.0 non-experts and GPT-3.5 non-experts for Experiment 1. The total number of questions categorized as Correct by each non-expert group is displayed.

|  | | Gpt-4.0 non-expert | | |
| --- | --- | --- | --- | --- |
|  |  | Correct | Other | Total |
| GPT-3.5 non-expert | Correct | 5 | 2 | 7 |
|  | Other | 28 | 59 | 87 |
|  | Total | 33 | 61 | 94 |
